# Scalable Manufacturing of Roll‐to‐Roll Slot‐Die Coated Perovskite Solar Cells

**DOI:** 10.1002/advs.202522103

**Published:** 2026-04-22

**Authors:** Farshad Jafarzadeh, MirKazem Omrani, Nathan S. Hill, Jazib Ali, Dumitru Sirbu, David G. Lidzey

**Affiliations:** ^1^ Unit 5, Jade Business Park Power Roll Ltd Seaham United Kingdom; ^2^ School of Mathematical and Physical Sciences The University of Sheffield Hicks Building, Hounsfield Road Sheffield United Kingdom

**Keywords:** ambient processing, perovskite solar cells, roll‐to‐roll processing, scalable fabrication, slot‐die coating

## Abstract

Perovskite solar cells (PSCs) have recently demonstrated laboratory‐scale power conversion efficiencies (PCEs) exceeding 27%, a value that now matches the most efficient silicon photovoltaics. Their low‐temperature, solution‐processable fabrication and compatibility with flexible substrates position PSCs as candidates for next‐generation photovoltaic technologies. Notably, PSCs fabricated on flexible substrates have been shown to demonstrate efficiencies that exceed 26% PCE, however the efficiency of flexible modules remains under 22%. Roll‐to‐roll (R2R) deposition has emerged as a leading candidate to transition PSC fabrication to a practical manufacturing environment. In this review, we focus on R2R processing of PSCs and discuss the techniques that have been developed to control perovskite structure, crystallinity, and morphology. In particular, we explore the effects of perovskite composition, solvent engineering, film‐drying, and additive engineering, which can be harnessed to optimise device performance, with fully R2R‐fabricated PSCs demonstrated having PCEs above 15%, and R2R printed modules reaching a PCE of 11%. A number of challenges remain, including maintaining film quality at high substrate throughput, ensuring the long‐term stability of devices, and reducing the cost of module integration.

## Graphical abstract

Roll‐to‐roll (R2R) slot‐die coating is emerging as a scalable pathway for perovskite solar cells. This mini‐review summarizes advances in ink design, drying control, and electrode integration that enable high‐efficiency flexible devices and modules. Challenges in throughput, stability, and sustainable processing are outlined, guiding the translation of laboratory results into industrial manufacturing.

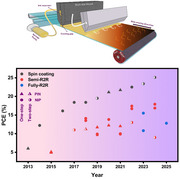

## 1 Introduction

The efficiency of organic–inorganic perovskite solar cells (PSCs) has advanced significantly since their initial report in 2009, when a power conversion efficiency (PCE) of 3.8% was first demonstrated [1]. Within just over a decade, laboratory‐scale PSCs have reached a PCE of 27.0%; a value only 0.3% lower than that of conventional silicon photovoltaics [2, 3]. This rise in performance is attributed to the unique optoelectronic properties of metal‐halide perovskites, including high absorption coefficient [4], long charge‐carrier diffusion lengths [5], high defect tolerance [6, 7], and tunable bandgaps [8, 9, 10, 11]. Beyond high efficiency, PSCs offer advantages over traditional silicon photovoltaics, such as low‐temperature and solution‐processability, together with compatibility with lightweight, flexible substrates [12]. Due to the high power‐to‐weight ratio and mechanical flexibility of PSCs, new applications are enabled [13, 14], such as wearable electronics [15, 16], integration with curved surfaces, and building‐integrated photovoltaics [17].

Translating the high performance of PSCs from the laboratory to commercial products requires scalable fabrication methods. These methods must enable manufacturing on a moving web with defined line speed and coating width, together with module‐relevant patterning and series interconnection strategies. Unlike silicon photovoltaics, which rely on batch wafer processing, PSCs can be produced using roll‐to‐roll (R2R) coating methods thanks to their ability to be processed from solution [18]. R2R processing is a continuous web‐based manufacturing method in which a flexible substrate is transported from an unwinder to a rewinder under controlled web tension and speed. Along the web path, sequential process modules such as coating, printing, drying, annealing, surface treatment, patterning, or lamination are integrated together, allowing functional layers to be deposited and transformed in a continuous manner. R2R production enables high‐throughput, low‐cost manufacturing of PSCs by printing them onto flexible plastic or metal foils [19, 20] and is commonly used in the printing and packaging industries [21]. The commercialization of PSCs will rely on closing the gap between the performance of large‐scale processed devices and small‐scale devices that are created in the laboratory by spin‐coating. Here, manufacturing strategy and cost competitiveness will depend on the desired product format and module architecture, for example, flexible vs rigid or single‐junction vs tandem [20, 22]. Possible manufacturing processes include sheet‐to‐sheet processing on glass and perovskite–silicon tandem integration, or R2R processing of flexible perovskite devices. For flexible PSCs, R2R manufacturing is a plausible route to high‐throughput fabrication and reducing production cost relative to batch processing [20, 23].

Fabrication of PSCs via R2R processing involves the sequential deposition of functional device layers onto a continuously transported flexible substrate, typically a transparent‐conductive‐oxide (TCO)‐coated polymer film, which is unwound from a feed roll and then rewound after processing. Among R2R‐compatible coating techniques used to process perovskite solar cells, slot‐die coating has emerged as the most widely adopted method for scale‐up due to its pre‐metered nature, compatibility with continuous processing, and ability to deliver uniform thin films over large areas [21, 24, 25, 26, 27, 28, 29, 30, 31, 32]. While alternative R2R techniques such as gravure printing have demonstrated high efficiencies on flexible substrates [33], the transfer‐based nature of gravure printing in which film formation depends on cell filling and ink transfer efficiency makes it less suitable for uniform large‐area multilayer deposition [34]. In contrast, slot‐die coating is a pre‐metered technique that provides deterministic control over wet film thickness.

**FIGURE 1:**
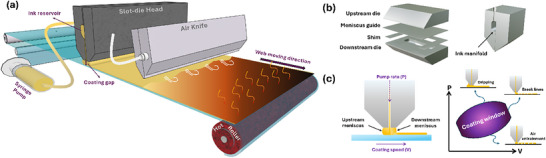
Schematics of a slot‐die coating line used in perovskite printing (a), dismantled slot die coater head (b), and the menisci positioning under different processing parameters and the coating window (c).

As shown in Figure 1a, slot‐die coating relies on delivering a solution through a narrow slit onto a moving substrate, which is then deposited as a wet film. Here, the film thickness and uniformity can be precisely controlled by adjusting the solution flow rate and substrate speed [35]. The slot‐die head that consists of different components, such as upstream and downstream die, shim, and meniscus guide (Figure 1b) that play a key role in even distribution of solution across the coating width and stabilizing the meniscus during the coating process [36]. The narrow channel confined between two dies of the slot‐die head is created by a shim that can control the pressure drop of the pumped solution passing through the channel. The pressure drop is determined by the Poiseuille flow equation, $\Delta P=\frac{12\mu LV}{b^{3}}\;$, where µ, V, L, and b are the solution viscosity, flow rate, the channel length, and width, respectively [37]. As the solution exits the slit, it creates a meniscus between the slot‐die head lips and the moving substrate. Due to the movement of the substrate, a various flow rates (known as Couette flow) is formed in the channel between the upstream/downstream lips and the substrate that is governed by the Navier–Stokes equation [38]. Here, the position of upstream and downstream menisci is determined by the balance between the Poiseuille and Couette flow dynamics, where the solution flow rate (i.e., pump rate) and coating speed govern them, respectively. Figure 1c illustrates how changing these processing parameters alters the menisci positioning and how it can deliver a stable coating window. This technique can also be used to produce patterned stripes using a shim or meniscus guide, allowing the fabrication of solar cell modules in which sub‐cells are separated by uncoated regions [29]. Furthermore, slot‐die coating offers efficient material usage and supports continuous, high‐speed processing on the meter‐per‐minute scale while maintaining uniform coatings. Despite such advantages, R2R slot‐die coating introduces new processing challenges compared to lab‐scale methods for PSC fabrication. In particular, perovskite crystallization differs significantly between spin coating and slot‐die coating due to a variation in solvent dynamics, nucleation mechanisms, and processing conditions [39]. This review outlines the rapid progress in R2R development of PSCs, emphasizing the need for scalable manufacturing to bridge the gap between laboratory research and industrial application. Table 1 summarises the key terminilogy of R2R perovskite manufacturing.

**TABLE 1:** Key terminology used in R2R perovskite manufacturing.

Term	Definition
Roll‐to‐roll (R2R)	Continuous manufacturing process in which a flexible substrate moves from an unwinder to a rewinder while passing through coating, printing, and drying stations.
Fully R2R fabrication	All device layers deposited using R2R‐compatible processes without batch deposition methods.
Hybrid R2R fabrication	A combination of R2R coating steps with batch processes, such as vacuum evaporation.
Slot‐die coating	Pre‐metered coating technique in which film thickness is controlled by flow rate and web speed.
Meniscus coating	Coating method in which a liquid meniscus is maintained between the coating head and substrate.
Single‐step deposition	Perovskite formed from a single precursor solution.
Two‐step deposition	Sequential formation of perovskite via metal‐halide precursor and organic salt conversion.
Module geometric fill factor	Ratio of active photovoltaic area to total module area.

## 2 Perovskite Crystallisation and Drying Dynamics

**FIGURE 2:**
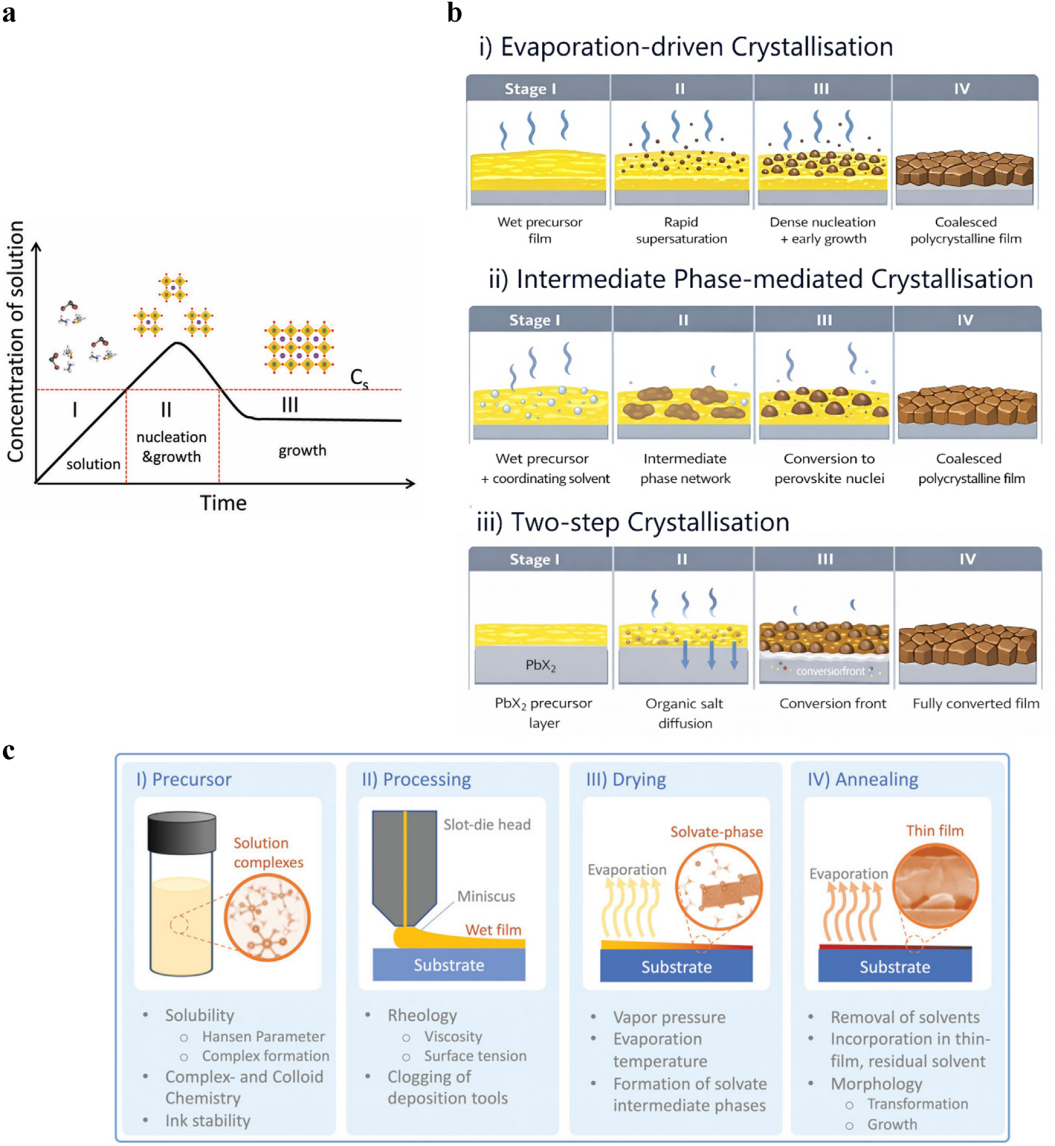
(a) The crystallisation stages of a perovskite based on the La Mer model. Reproduced with permission from [40] Copyright 2018 John Wiley and Sons. (b) Schematics of crystallization pathways of perovskite film in slot‐die coating. (c) The processing stages of thin films by solution‐based methods, such as slot‐die coating. Reproduced with permission from [46]. Copyright 2023 John Wiley and Sons.

The crystallization of a perovskite from a precursor solution can be explained using classical nucleation theory and the La Mer model [40, 41]. The La Mer model consists of three main stages, as shown in Figure 2a. In the first stage, solvent evaporation results in an increase in the precursor concentration, with the solution reaching a critical supersaturation concentration (C_s_). At this point, “stage II” is reached in which rapid nucleation and growth of perovskite crystals occur. The precursor solution is at this point in a non‐equilibrium state, with supersaturation acting as the driving force for crystal nucleation. The nuclei, which form, then grow following the diffusion of precursors from the surrounding solution. At some point within stage II, the solution reaches its maximum concentration (C_max_) due to a trade‐off between the rate at which the solvent is evaporating and the “consumption” of the monomers due to crystal nucleation and growth. At this point, the solution concentration then starts to reduce, and at some point, falls below C_S_. Nucleation then stops, and the process enters “stage III” in which the growth of crystals saturates, with solution concentration eventually reaching an equilibrium level.

On the basis of the La Mer model, one should expect the formation of uniform, densely packed, and defect‐free perovskite films to require rapid nucleation and slow crystal growth [41]. In a spin coating process, the centrifugal force aids solvent evaporation and drives the high evaporation rate necessary to create a high density of nuclei. However, rapid crystal growth can result in an imbalance between nucleation and growth, resulting in the development of a dendritic perovskite structure. Antisolvents that are miscible with the primary solvent but do not dissolve the precursors can be applied to a wet film during spin coating to induce nucleation and rapid supersaturation. This is generally followed by thermal annealing of a perovskite film to evaporate any remaining solvent and increase the diffusion rate, resulting in accelerated grain growth.

In contrast to spin coating, meniscus‐coating deposition techniques such as slot‐die coating and doctor blading form films via a dynamic meniscus that transfers liquid to the substrate, governing the wet‐film thickness and solvent removal profile. However, slot‐die coating operates as a pre‐metered process in which thickness is primarily set by the liquid flow rate and substrate speed, whereas doctor blading is a self‐metered process that typically follows a Landau–Levich regime, with thickness determined by the balance of viscous drag and capillary forces [42, 43]. In these approaches, solvent evaporation is typically slower than in spin coating because of the absence of centrifugal force. For this reason, film drying is often accelerated using one or a combination of a number of methods, such as hot casting [44], gas quenching [45], the use of volatile solvents [46], or vacuum quenching [47]. Both doctor blading and slot‐die coating belong to the broader class of meniscus‐coating methods and therefore share similar wet‐film formation and evaporation‐driven supersaturation dynamics, with grain growth being governed by similar physicochemical principles [42, 48]. Note, however, slot‐die coating introduces additional process constraints, for example, the requirement to balance ink flow rate with web speed and maintain a stable coating bead. Chen et al. compared perovskite crystallisation using spin coating and doctor blading, and found that although both methods exhibit crystallisation that starts at the film surface, the underlying mechanisms differ [49]. In spin coating using an antisolvent treatment, top‐down grain growth occurs in two stages: a rapid conversion within the first few seconds, followed by a slower process lasting up to 10 min. It was suggested that the film surface crystallises quickly during annealing, while residual solvents such as Dimethyl sulfoxide (DMSO) take longer to escape from the bulk. In contrast, blade coating using gas quenching and a volatile solvent system initially forms a mixed phase of perovskite and other intermediate compounds at room temperature. Such remaining intermediate phases are then eliminated upon thermal annealing. The study also concluded that solvent evaporation from the top surface initiates vertical crystallisation in blade‐coated films. It seems, therefore, that solvent drying is a critical step necessary to create high‐quality perovskite films.

In slot‐die coating, perovskite crystallization can be explained through three distinct pathways defined by how supersaturation and phase conversion are induced during the drying of a wet film, as depicted in Figure 2b. One widely adopted route is evaporation‐accelerated nucleation, in which rapid solvent removal via gas‐quenching, hot casting, vacuum quenching, or the use of high‐volatility solvent systems drives rapid supersaturation and high nucleation density, enabling continuous film formation. However, if the solvent quench is excessively rapid, grain growth becomes kinetically limited, suppressing Ostwald ripening and preventing the dissolution of smaller crystallites into larger grains [50]. A second route is intermediate‐phase mediated crystallization, where highly coordinating solvents/additives such as dimethyl sulfoxide (DMSO), N‐Methyl‐2‐pyrrolidone (NMP), and Dipropyl sulfoxide (DPSO) are used to stabilize solvent–perovskite adducts that subsequently convert to the perovskite phase during annealing. This allows a decoupling of coating and crystallization but introducing sensitivity to residual solvent and incomplete conversion [51, 52, 53]. A third pathway involves sequential layer deposition, in which a metal‐halide precursor (PbX_2_) layer is first deposited and later converted to a perovskite by exposure to organic halide salts by diffusion‐limited conversion. This can offer improved tolerance to ambient processing but is susceptible to incomplete conversion or the formation of residual PbX_2_ if diffusion and reaction kinetics are not well balanced [24, 44].

The solution‐processed deposition of perovskites is highly dependent on the casting solvent that is used. One study categorized the role of the solvent in the different stages of perovskite film growth as depicted in Figure 2c [46]. When selecting a precursor‐solvent (precursor), a high solubility of the various components is necessary at the desired molar concentrations. In the second stage (processing), solvents play a critical role in achieving a uniform wet film during coating by tuning the rheology and surface tension of the ink. Stage III (drying) involves the controlled removal of solvent; a process that promotes thin‐film formation and may lead to the formation of intermediate phases. Finally, in the last stage (thermal annealing) a conversion to the crystalline perovskite phase happens through the removal of all remaining solvent.

One of the main challenges in controlling the crystallization of a perovskite during R2R slot‐die coating is to adjust the solvent evaporation rate via thermal annealing to create a high‐quality film. As discussed above, spin coating commonly relies on anti‐solvent treatments to induce rapid crystallization, a step that cannot be applied in R2R slot‐die coating [30]. In contrast, R2R slot‐die coating requires precise control of drying kinetics, ink rheology, and process parameters to create high‐quality films comparable to those made under laboratory conditions. Each coated film must dry rapidly, as prolonged crystallisation or annealing will interrupt a continuous process or will require equipment having a large physical footprint. Furthermore, the flexible substrates used in R2R systems cannot tolerate high temperatures (typically above 140°C); a constraint that further limits thermal treatment options [30]. For this reason, achieving uniform perovskite crystallization at high line speeds is particularly challenging. Perovskite inks often crystallize quickly, with the films produced containing defects such as pinholes or incomplete coverage if the deposition process is not properly managed. While gas quenching combined with R2R processing can be effective, it requires fine‐tuning and may suffer from reproducibility issues in large‐scale processes due to factors like airflow turbulence or limitations in system configurations [54, 55]. However, when fully optimized, gas quenching can completely replace antisolvent steps in a continuous process line. Key components in the parameter space optimization include gas flow rate, air‐knife angle, air‐knife slit opening size, and air‐knife to web distance [55].

## 3 Recent Progress in R2R Slot‐Die Coating of PSCs

### 3.1 Perovskite Composition

A variety of perovskite absorber materials have been adapted for R2R fabrication, with these materials designed to improve device performance and stability. Early R2R demonstrations often used methylammonium lead triiodide (MAPbI_3_) perovskite. For example, Zuo et al. formulated a methylammonium lead iodide ink with ammonium chloride (NH_4_Cl) additive to control crystallization, achieving high‐quality MAPbI_3_ films [31]. Using this formulation, a lab‐scale PCE of 19.5% in ambient air was attained, with the process then translated to slot‐die coating, reaching a PCE of 15.6% on glass, and around 11.2% PCE on a flexible substrate using R2R coating.

Following this, mixed‐cation perovskites (i.e., incorporating formamidinum (FA) and cesium (Cs) in addition to methylammonium (MA) in the precursor solution) and mixed‐halide (i.e., a mix of iodide and bromide anions) perovskites emerged, having improved stability both at elevated temperature and under ambient conditions. For example, Othman et al. demonstrated a triple‐cation mixed‐halide perovskite (FA_0.79_MA_0.15_Cs_0.06_Pb(I_0.85_Br_0.15_)_3_) processed by R2R slot‐die in an inverted cell architecture, achieving a PCE of 12% [56]. Others then employed FA‐rich compositions and 2D/organic additive strategies to enhance moisture resistance when coating in ambient air [21, 26]. Recently, a bromide, wide‐bandgap perovskite (CsFAPbBr_3_) having a bandgap of 2.3 eV has been explored using R2R printing. This formulation directly targeted building‐integrated and tandem applications [24].

We note that the materials used in R2R PSCs come from the same family of halide perovskites as those used in high‐efficiency spin‐coated cells. However, it is necessary to tailor perovskite ink formulations for compatibility with R2R processing; this involves incorporating features such as humidity‐tolerant polymers, alternative solvents, and crystallization additives.

### 3.2 Performance of R2R Slot‐Die Coated PSCs

**FIGURE 3:**
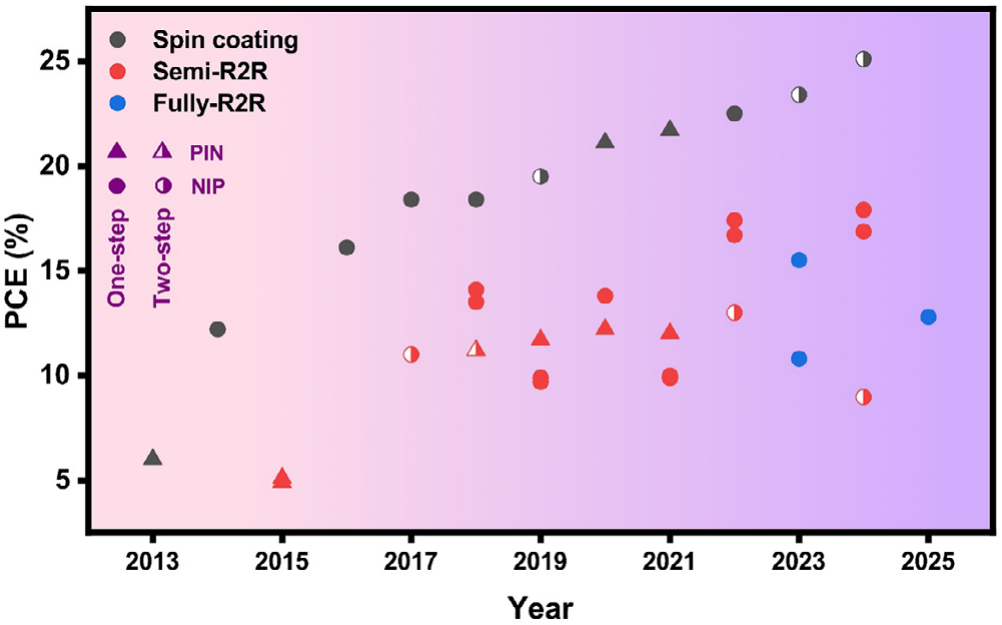
PCE trends of R2R processed perovskite solar cells compared to small‐scale devices fabricated by spin coating [2, 24, 25, 26, 27, 28, 29, 30, 31, 32, 33, 39, 44, 47, 56, 57, 58, 59, 60, 61, 62, 63, 64, 65].

The power conversion efficiencies of R2R‐fabricated PSCs have steadily improved, closing the gap with spin‐coated devices. Figure 3 compares the evolution in efficiency over the last years of R2R printed PSCs with the state‐of‐the‐art spin coated devices.

#### 3.2.1 Single‐Step Deposition

**FIGURE 4:**
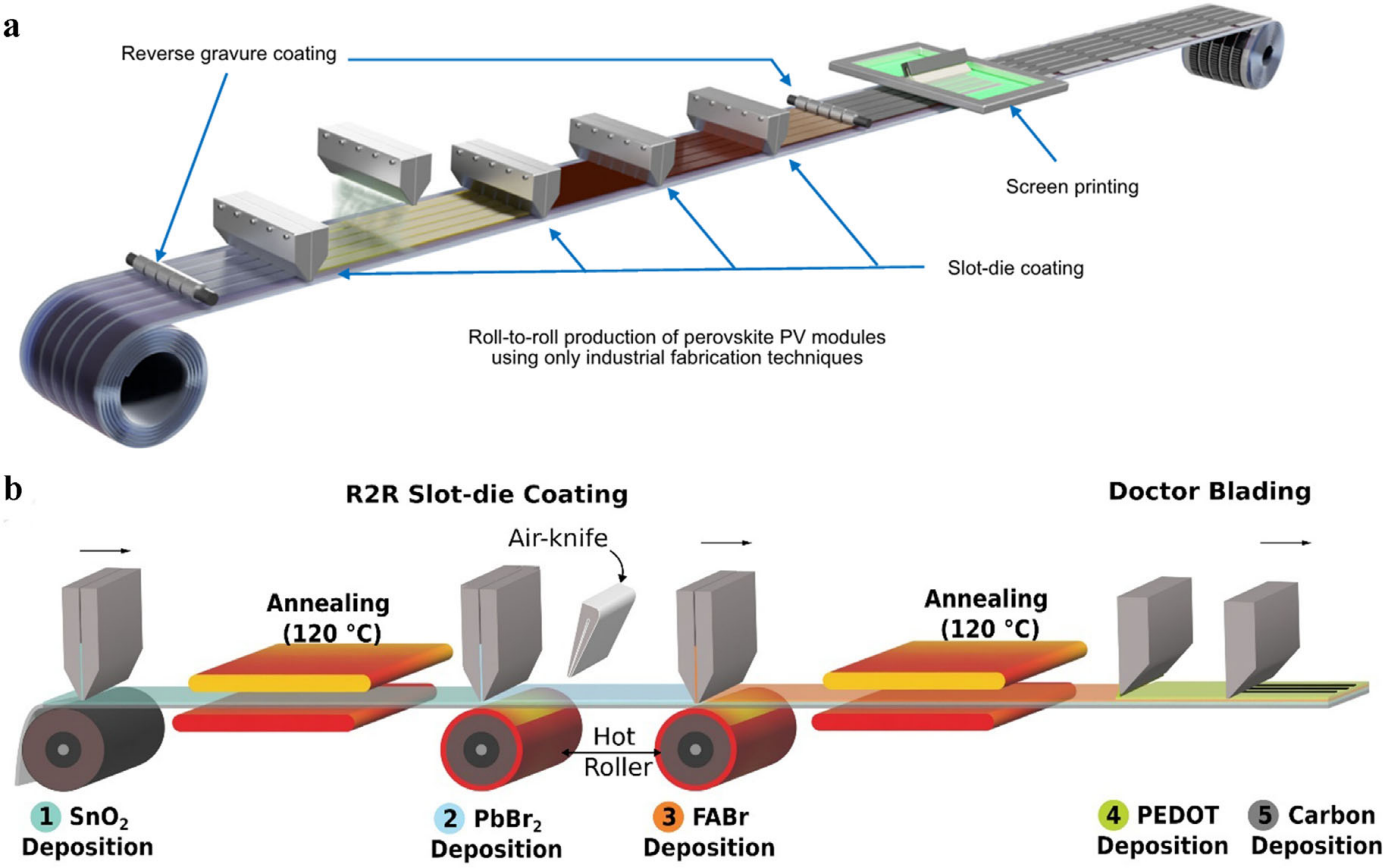
(a) A schematic of a fully R2R perovskite solar cell production based on a single‐step perovskite deposition process reproduced from [26] under the terms of the Creative Commons Attribution (CC BY) license. (b) A schematic representation of a two‐step R2R perovskite deposition process. Reproduced with permission from. [24] Copyright 2024 John Wiley and Sons.

The first demonstrations of R2R slot‐die coated PSCs were reported in 2015, with PCEs of between 4.9% and 5.1% attained, with all layers except the back electrode deposited using R2R coating methods [62, 63]. Single‐step R2R PSCs demonstrated a PCE of 11.16% in 2018 by introducing NH_4_Cl additive to MAPbI_3_, which enhanced film morphology [31]. Galagan et al. then demonstrated an efficiency of 13.5% by using R2R to coat the MA‐free perovskite Cs_0.15_FA_0.85_PbI_2.85_Br_0.15_. A triple cation perovskite device with a PCE of 16.7% was then demonstrated by Sutherland et al. that incorporated a dry‐pressed Ag/carbon electrode [28]. This was comparable in efficiency to a control device that had an evaporated gold electrode, which had a PCE of 17.4%; a result demonstrating a minimal efficiency loss when moving to a fully printed approach. Notably, Weerasinghe et al. reported fully R2R‐coated PSCs coated in an ambient environment utilizing a R2R printable carbon paste and achieved a PCE of 15.5%. Modules having an active area of 50 cm^2^ and a PCE of 11% were also demonstrated [26]. A schematic of the full R2R production of these PSCs is shown in Figure 4a. Here, the electron transport layer (ETL), perovskite, and hole transport layer (HTL) were deposited by R2R slot‐die coating, with the carbon electrode being screen printed.

While traditional perovskite precursor inks have generally relied on the use polar aprotic solvents such as N,N‐dimethylformamide (DMF) and DMSO), Dou and colleagues reported MAPbI_3_ devices deposited from an acetonitrile solvent that had been exposed to a methylamine gas. Such films dry quickly and form uniform films without the need of gas quenching or hot casting. Using this material system, R2R‐coated PSCs on flexible glass substrates were reported having a PCE of 14.1% [60]. Burkitt et al. used a similar solvent system on a polyethylene terephthalate (PET) substrate, achieving devices with a PCE of 12.2% thanks to the introduction of an HCl additive into the perovskite precursor. Film drying was also controlled using a gas quenching technique [29].

#### 3.2.2 Two‐Step Deposition

The two‐step deposition method was originally developed for spin coating, but has been effectively adapted for blade coating, making it suitable for large‐scale commercial applications [66, 67]. This method involves initially depositing a layer of lead iodide (PbI_2_) on a substrate, followed by converting it into a perovskite phase through exposure to a solution containing organic halide salts. The two‐step deposition technique significantly enhances the fabrication of high‐quality perovskite films and is especially useful in environments with fluctuating humidity levels [58, 68]. In 2017, Heo et al. reported devices with a 11% PCE processed via R2R slot‐die coating in air using sequential deposition; here, PbI_2_ was first deposited, followed by gas quenching and methylammonium iodide (MAI) deposition [32]. It was shown that film processability was improved by retarding the crystallisation and aiding the perovskite phase formation by adding less than a stoichiometric amount of organic salt (30 mol% of MAI) to the second step solution.

Park and colleagues then explored a two‐step deposition of a CsFAPbI_3_ perovskite by incorporating Cs salts in the first step, followed by formamidinium iodide (FAI) deposition in the second step. With the combination of intensive pulse light annealing (IPL), together with the addition of a Cs formate salt in the PbI_2_ solution, a device PCE of 16.87% was demonstrated, with 10 cm × 10 cm perovskite solar modules reaching a PCE of 11.25% [21]. Jafarzadeh et al. also demonstrated that two‐step R2R slot‐die coating can be used to deposit wide‐bandgap perovskites using bromide‐only salts in an ambient environment [24]. The incorporation of CsBr in the PbBr_2_ solution, together with the addition of 10% butanol, enabled the fabrication of PSCs having a PCE of 8.97%, with 5 × 5 cm^2^ modules created having a PCE of 5.8%. Figure 4b shows a schematic of the two‐step R2R deposition of a CsFAPbBr_3_ perovskite.

Overall, both single‐step and two‐step R2R deposition methods are effective for scalable fabrication of perovskite solar cells, though each presents distinct trade‐offs. The single‐step approach offers a simpler, faster process that supports continuous printing and accommodates diverse solvent and additive systems. It has achieved the highest reported PCEs for R2R‐processed devices, reaching nearly 18% for small‐area cells and over 11% for modules [21, 26]. In contrast, the two‐step method provides finer control over crystallization and film uniformity, making it suitable for compositional tuning and for wide‐bandgap or mixed‐halide perovskites, though efficiencies are typically lower. From a manufacturing perspective, these trends suggest that single‐step deposition strategies combined with solvent and drying engineering are currently more compatible with high‐throughput, fully inline R2R processing, as they minimize process complexity and reduce the number of sequential coating and conversion steps. In contrast, the additional processing stages required in two‐step deposition introduce challenges for integration into continuous production lines, particularly at high web speeds.

The key performance metrics and fabrication details of reported R2R slot‐die coated perovskite solar cells and modules are summarized in Tables 2 and 3, respectively. Note that in these tables, R2R‐coated perovskite solar cells and modules are classified as being Hybrid R2R when the perovskite stack is only partly deposited by R2R processing, with at least one key layer fabricated off‐line (most often the vacuum‐deposited metal electrode). In contrast, Fully R2R refers to devices in which all functional layers are deposited in an R2R‐compatible in‐line sequence, enabling in‐line drying or curing, including electrode formation by printing, lamination, or vacuum web coating. Tables 2 and 3 highlight that the highest device efficiencies are predominantly achieved in hybrid R2R architectures that still rely on vacuum‐deposited electrodes, whereas fully R2R devices although are rapidly improving generally exhibit lower efficiencies, underscoring that electrode integration and interface control remain key bottlenecks for fully inline manufacturing.

**TABLE 2:** Summary of reported R2R slot‐die coated perovskite solar cells, showing the year, device architecture, perovskite and electrode deposition methods, and corresponding PCE.

Year	Device Architecture	Perovskite Deposition	Electrode	PCE (%)	Refs.
Hybrid R2R					
2015	PET/ITO/PEDOT:PSS/MAPb(ICl)_3_/PCBM/ZnO/Ag	Single step	Ag (Screen Printing)	4.9	[63]
2015	PET/ITO/PEDOT:PSS/MAPb(ICl)_3_/PCBM/ZnO/Ag	Single step	Ag (Evaporation)	5.1	[62]
2017	PET/ITO/ZnO/FAMAPbI_3_/PEDOT/MoOx/Ag	Two‐step	Ag (Evaporation)	11	[32]
2018	PET/ ITO/PEDOT:PSS/ MAPbI_3_/PCBM/Ca/Al	Two‐step	Al (Evaporation)	11.2	[31]
2018	PET/ITO/SnO_2_/CsFAPb(IBr)_3_/Spiro/Au	Single step	Au (Evaporation)	13.5	[61]
2018	Flex Glass/ IZO/SnO_2_/MAPI/Spiro/Au	Single step	Au (Evaporation)	14.1	[60]
2019	PET/ITO/PEDOT:PSS/MAFACsPb(IBr)_3_/PCBM/C60/PEIE/Ag	Single step	Al (Evaporation)	11.7	[30]
2019	PET/ITO/ SnO_2_/MAFACsPbI_3_/Spiro/Ag	Single step	Ag (Evaporation)	9.9	[69]
2020	PET/ PEDOT:PSS/MAPI/PCBM/BCP/Ag	Single step	Ag (Evaporation)	12.2	[29]
2020	PET/ITO/SnO2/FAMAPb(IBr)_3_/P3HT/Au	Single step	Au (Evaporation)	13.8	[33]
2021	PET/ITO/PEDOT:PSS/CsFAMAPb(IBr)_3_/PCBM/PEIE/Au	Single step	Au (Evaporation)	12.0	[56]
2021	PET/ITO/SnO_2_/FAMAPbI_3_/PPDT2FBT/C/Cu/Al	Single step	C/Cu/Al (Pressure Lamination)	10.0	[59]
2022	PET/ITO/SnO_2_/CsFAMAPb(IBr)_3_/ Spiro/C/Ag	Single step	Au (Evaporation)	17.4	[28]
2022	PET/ITO SnO_2_/ MAPbI_3_/Spiro/Ag	Single step	Ag (Evaporation)	13.0	[44]
2022	PET/TCE/SnO_2_/CFAMPb(IBr)_3_/Spiro/carbon/Ag	Single step	Ag/C (Bar Coated‐Pressed)	16.7	[28]
2023	PET/ITO/SnO_2_/ FAMAPbI_3_/PPDT2FBT/Au	Single step	Au (Evaporation)	17.9	[26]
2024	PET/IMI/SnO_2_/CsFAPbBr_3_/PEDOT/Carbon	Two‐step	Carbon (Blade Coating)	8.9	[24]
2024	PET/ITO/SnO_2_/CsFAPbI_3_/Spiro/Au	Single step	Au (Evaporation)	16.9	[21]
Fully R2R					
2023	PET/ITO/SnO_2_/ MAPbI_3_/PEDOT/Carbon	Single step	Carbon (R2R)	10.8	[27]
2024	PET/ITO/SnO_2_/FAMAPbI_3_/PPDT2FBT/Carbon	Single step	Carbon (R2R)	15.5	[26]
2025	Ti/SnO_2_/C60/ MAPbI_3_/NiO/Ni	Single step	R2R Vacuum Coating	12.8	[25]

**TABLE 3:** Reported R2R printed perovskite solar modules with the year, device architecture, perovskite deposition method, and corresponding module area and PCE.

Year	Device Architecture	Perovskite Deposition	Module Area (cm^2^)	Module PCE (%)	Refs.
Hybrid R2R					
2024	PET/IMI/SnO_2_/CsFAPbBr_3_/PEDOT/Carbon	Two‐step	16.8	5.8	[24]
2024	PET/ITO/SnO_2_/CsFAPbI_3_/Spiro/Au	Single step	94.6	11.3	[21]
Fully R2R					
2024	PET/ITO/SnO_2_/FAMAPbI3/PPDT2FBT/Carbon	Single step	50.0	11.0	[26]

### 3.3 Device Stability

Stability is a critical concern for PSCs, and it becomes even more important when scaling devices up for manufacture. For R2R‐fabricated PSCs, achieving long‐term operation means addressing both intrinsic material stability and extrinsic factors such as moisture ingress, thermal cycling, and mechanical stress on flexible modules.

Advances in perovskite compositions and interfaces have led to the development of more robust R2R‐printed cells. The move from pure MAPbI_3_ to mixed cation formulations has increased both thermal and phase stability. In one of the first stability measurements of R2R‐coated PSCs, a fully printed flexible device incorporating a carbon electrode showed negligible efficiency loss after 24 h of continuous 1‐sun illumination when encapsulated using a barrier film. This initial level of operational stability was attributed to the inert and robust carbon back electrode as well as the use of stable interfaces that did not degrade under light [28]. Beynon et al. then showed that fully R2R‐coated perovskite cells with PET/ITO/SnO_2_/perovskite/PEDOT/carbon stack retained 84% of their original efficiency after 1000 h at 70% relative humidity and at 25°C in the dark [27]. Table 4 summarizes the reported stability of R2R‐processed perovskite solar cells.

**TABLE 4:** Reported stability of R2R processed Perovskite solar cells.

Protocol	Device Architecture	Stability	Refs.
ISOS D1 (not specified)	PET/ITO/PEDOT:PSS/MAPb(ICl)/PCBM/ZnO/Ag	T_50_ (100 h)	[62]
ISOS D1 (not specified)	PET/ITO/SnO2/FAMAPb(IBr)3/P3HT/Au	∼T_99_ (960 h)	[33]
ISOS D1 (not specified)	PET/IMI/SnO_2_/CsFAPbBr_3_/PEDOT/Carbon	T_87_ (4,000 h)	[24]
ISOS D1 (RT, 10% RH)	PET/ITO/SnO_2_/FAMAPbI_3_/PPDT2FBT/C/Cu/Al	T65 (2,400 h)	[56]
ISOS D3 (65°C, 85% RH)	PET/IMI/SnO_2_/CsFAPbBr_3_/PEDOT/Carbon	T_80_ (125 h), T_40_ (350 h)	[24]
ISOS D3 (65°C, 85% RH)	PET/ITO/SnO_2_/MAPI/PEDOT/Carbon	T_83_ (3,600 h)	[27]
∼ISOS L1 (not specified)	PET/ITO/PEDOT:PSS/CsFAMAPb(IBr)_3_/PCBM/PEIE/Au	T_95_ (33 h)	[56]
ISOS L1 (ambient, 0.7 sun)	PET/IMI/SnO_2_/CsFAPbBr_3_/PEDOT/Carbon	T_80_ (550 h)	[24]

Mechanical stability is also important, since R2R modules must withstand bending and handling during their production. Additional mechanical robustness is also required if the product is intended to be bent and or stretched during operation. Sutherland et al. demonstrated that a device architecture of PET/ITO/SnO_2_/CsFAMAPb(IBr)_3_/Spiro‐OMeTAD/C/Ag with printed‐carbon/silver electrode could be bent 3000 times around a bending radius of 10 mm while retaining >90% of its initial efficiency [28]. This mechanical robustness is partly due to the avoidance of thick metal electrodes, together with the use of conductive carbon composites and polymer‐based layers that can flex without cracking. Such durability is essential for applications like wearable PV or roll‐up solar chargers. It also suggests that R2R fabrication (which involves winding and unwinding flexible substrates) does not introduce fatal mechanical stress into the device stack.

We note that although R2R PSC devices have shown encouraging stability under dark storage, damp‐heat tests, and bending experiments, these results should not be interpreted as evidence of achieving commercial readiness. Achieving levels of stability similar to silicon‐PV (25+ years) remains a major hurdle for PSCs, especially for R2R‐coated devices. Indeed, ensuring stability under real‐world outdoor conditions remains a challenge. Here, a key reason for this is that many indoor stability tests do not reproduce the combined stressors experienced outdoors, including ultraviolet irradiation, oxygen ingress, light and temperature cycling, reverse‐bias stress under partial shading, and module‐level degradation that is often initiated at interconnections and encapsulation weak points [70]. For large‐area perovskite modules in particular, dominant outdoor degradation pathways include photooxidation, ion migration, and interfacial reactions under thermal‐light stress, delamination and adhesive failure at scribed or laminated regions, and encapsulation failure accelerated by UV exposure and thermomechanical cycling [70]. It is clear therefore, that future progress should therefore be evaluated not only by International Summit on Organic Photovoltaic Stability (ISOS) metrics, but also by outdoor‐relevant multi‐stressor testing that captures module‐level failure propagation. With further refinements, R2R PSC modules are expected to meet the longevity requirements for practical deployment, especially when encapsulated using flexible barrier materials.

## 4 Challenges in R2R Slot‐Die Coated PSCs

### 4.1 Controlled Film Formation

Replicating the high‐quality perovskite films found in spin‐coated cells in a fast R2R process is a significant challenge. Here, the continuous coating process imposes a number of constraints; for example, wet films must crystallize quickly without the use of anti‐solvent quenching steps. Furthermore, there is limited time for the solvent to evaporate on a moving substrate. If not carefully managed, this can lead to incomplete conversion or the formation of morphological defects (e.g., pinholes, non‐uniform sized grains) that reduce device efficiency. A combination of heated stages and the use of gas quenching techniques were introduced to accelerate solvent evaporation and induce nucleation and crystal growth in perovskite wet films. The quenching gas flow rate, its temperature, and substrate temperature play key roles in achieving high‐quality perovskite films over large areas [55, 66]. By engineering the solvent system, optimizing coating and quenching parameters, and introducing additives to the perovskite precursor ink, researchers have been able to control crystallization kinetics of the perovskite [71]. For example, mixing non‐volatile coordinating solvents (e.g., DMSO) with volatile non‐coordinating solvents (e.g., acetonitrile, 2‐methoxyethanol) enabled Yehao et al. to deposit blade‐coated modules at a high deposition speed (99 mm/s) having 16.4% PCE with an area of 60 cm^2^ [72]. Kim et al. added 0.2 wt.% of polyethylene oxide (PEO) to a perovskite precursor to retard crystallization. By hot‐casting the film, it was possible to significantly increase the tolerance of the wet film to ambient humidity, with dense, uniform crystalline films produced upon drying [30]. The addition of Cs salts such as CsI, CsBr, and Cs formate have also been shown to enhance perovskite crystallinity by aiding the formation of phase‐pure perovskite [21, 24, 44]. Adding butanol as a co‐solvent has also been shown to improve perovskite film quality by lowering surface tension [24].

In summary, by using chemical additives and engineering controls, the challenge of fast yet controlled film formation in R2R processes is being addressed. This has been shown to yield perovskite layers having comparable quality to those made by slower batch processes.

### 4.2 Ambient Processing Challenges

Perovskites are sensitive to moisture and oxygen during film formation, however high‐performance cells are traditionally made in inert gloveboxes or dry rooms. For practical, scalable, and commercial R2R production, coating in ambient air is desirable to avoid costly environmental controls. Beyond the use of ink additives such as polyethylene oxide (PEO) that confer humidity tolerance [30], researchers have shown that certain perovskite compositions can be processed under ambient conditions with devices having minimal efficiency loss. For instance, wide‐bandgap perovskites rich in bromide have been found to be less moisture‐sensitive and have been successfully coated in air [24]. High‐throughput experimentation has also helped define robust process windows. For example, Weerasinghe et al. performed a combinatorial R2R trial of 1600 devices processed under different conditions to identify a parameter space that allowed high‐quality films to be created, even under ambient conditions [26]. By systematically optimizing coating speed, drying temperature, and ink chemistry, solar cells were created under ambient conditions with a PCE of 15%. This demonstrates that with proper precursor formulation and process tuning, R2R PSC fabrication can be made compatible with ambient processing. This finding should greatly simplify the R2R scale‐up process.

### 4.3 Electrode Deposition and Module Layout

The metal back electrode (e.g., gold or silver) in high‐efficiency PSCs is typically deposited by thermal evaporation, a vacuum process that is clearly very different from R2R solution‐based coating. The cost of gold is very high and can account for 70% of the module cost in lab‐scale devices [73, 74]. Additionally, connecting multiple cells in series on a flexible module requires patterning via laser scribing steps that need adaptation for R2R production.

Carbon‐based conductive inks and pastes have emerged as a viable alternative to evaporated metals, offering good conductivity and stability at a fraction of the cost [64]. Such carbon electrodes can be doctor‐bladed, or screen printed under ambient conditions. Sutherland et al. demonstrated a vacuum‐free, solvent‐free lamination method to deposit a carbon electrode onto printed PSC films, yielding flexible R2R devices with record PCEs of 16.7% reported [28]. Beynon et al. similarly used a solution‐processed carbon top electrode in a fully R2R all‐printed architecture [27]. Replacing ITO is an active area of research, with novel architectures such as back‐contact perovskites explored to eliminate transparent electrodes entirely. Blackburn et al. recently fabricated back‐contact perovskite mini‐modules on flexible sheets using fully R2R methods, with embossed micro‐grooves having alternating walls coated with *n*‐ and *p*‐type contacts [25]. In this approach, the electrodes and charge transport layers were deposited via R2R vacuum coating, while the perovskite layer was deposited using R2R slot‐die coating. The back‐contact architecture that was used eliminated the need for top electrodes, transparent conductive oxides, and laser processing, simplifying the device structure and reducing costs [25]. Such fully printed groove devices achieved PCEs up to 12.8%, and importantly, contained no indium or other expensive materials, suggesting a route toward a sustainable, low‐cost module technology.

**FIGURE 5:**
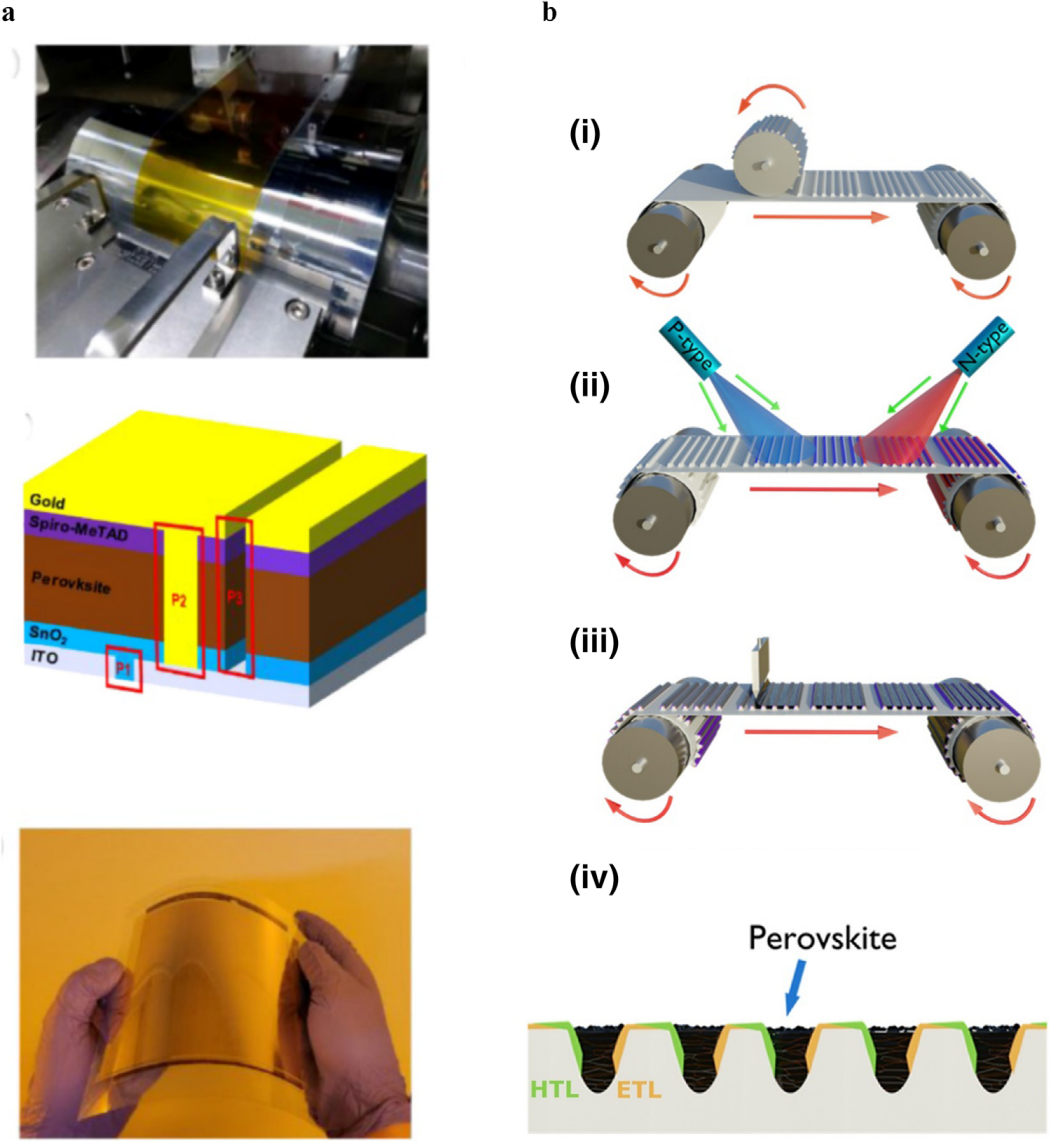
(a) Schematic and photograph of conventional R2R slot‐die coated modules with P1‐P2‐P3 laser scribing, having a dimension of 10 × 10 cm^2^. Figures reproduced with permission from [21]. Copyright 2024 American Chemical Society. (b) Schematic of groove‐based architecture perovskite module production (i) embossing, (ii) deposition of charge transport layers, (iii) perovskite deposition and (iv) schematic of device cross‐section reproduced from [25] under the terms of the Creative Commons Attribution (CC BY) license.

For module integration, laser or mechanical scribing used in rigid modules can be translated to R2R processing by in‐line patterning. For instance, laser scribers on a roll system have been used to create the P1, P2, and P3 isolation lines as shown in Figure 5a. Some R2R lines have incorporated such scribing steps between coatings, or have used masked printing to create stripes to achieve series connection [24, 26]. For the back‐contact groove devices reported by Blackburn et al., series connection was achieved through the inherent geometry of the device design, with each filled groove being series connected to its neighbour without use of laser ablation [25]. Figure 5b highlights this back‐contact groove design, with the corresponding photograph showing a module having such a device architecture.

### 4.4 Scalability and Cost

**FIGURE 6:**
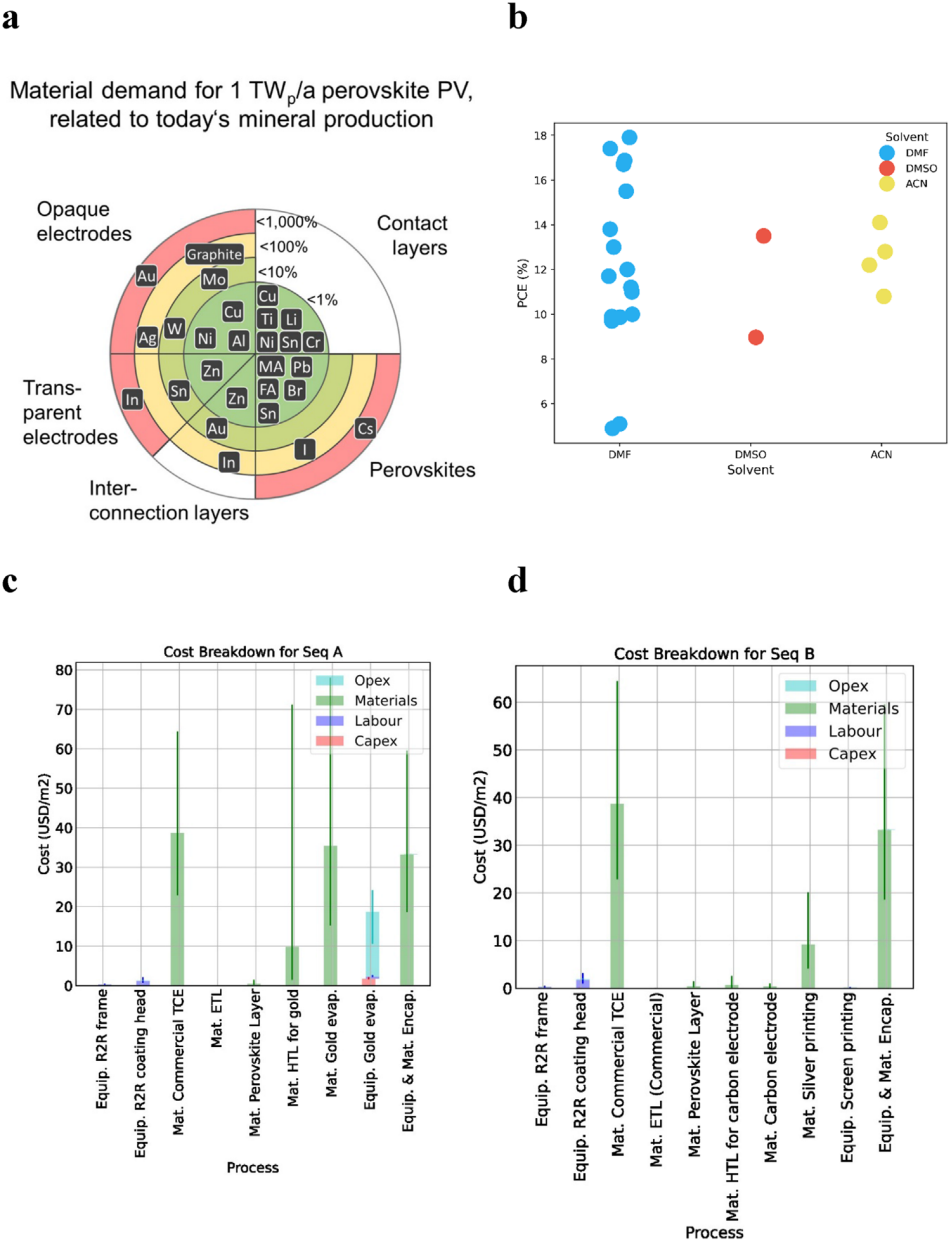
(a) Supply risk assessment of inorganic materials used for TW‐scale perovskite PV. Reproduced with permission from [75]. Copyright 2024 Elsevier. (b) Solvents used in the R2R‐processed PSCs versus device performance. (c) Projected production cost for Au electrode‐based modules and (d) Projected production cost for printed carbon electrode‐based modules reporoduced from [26] under the terms of the Creative Commons Attribution (CC BY) license.

Beyond the technical aspects discussed above, scaling the manufacture of modules to millions of square meters introduces economic and supply chain challenges. Wagner et al. conducted a quantitative assessment of material demand for multi‐terawatt (TW) scale perovskite tandem PV production and found that while most materials used in PSCs are not supply‐limited, a few key materials pose serious scalability challenges, as shown in the Figure 6a [75]. Indeed, it was emphasized that while perovskite layers use low material volumes due to their sub‐micron thickness, the cumulative material demand for multi‐TW annual production would be non‐negligible. For example, producing 1 TWp of perovskite tandem modules would require over 30 000 tons of material distributed across all functional layers. Substituting high‐risk materials is not only possible but necessary. For transparent electrodes, materials such as Aluminium doped Zinc Oxide (AZO) offer viable paths to eliminate indium are being explored but no commercial solution is currently available [76]. Similarly, for opaque electrodes, graphite and base metals like copper or aluminium are being considered as replacements for gold and silver.

We note that a techno‐economic analysis of R2R fabricated PSCs indicates that total module manufacturing cost is dominated by materials rather than by coating equipment [77]. The major cost drivers in PSCs include transparent conductive oxides, organic transport layers, and vacuum‐deposited rear electrodes [22]. Bottom‐up uncertainty‐based cost modelling of R2R processed PSCs showed that replacing evaporated metals with printed contacts and adopting lower‐cost active‐layer stacks can reduce median manufacturing cost to $37 m^−2^, whereas further increases in web speed provide cost returns. A recent cost analysis by Weerasinghe et al. estimated that, at a production volume of 1 000 000 m^2^ / year, printed perovskite modules could be manufactured for 0.7$/W, including both materials and processing. The projected production cost breakdown of devices made by two different processes (sequences) are shown in Figure 6c,d. Here, devices respectively contain many of the same layers but utilize Au as the electrode (sequence A) compared with a carbon electrode (Sequence B). It can be seen that the elimination of gold and the minimization of indium content result in a dramatic drop in material costs. Indeed, devices utilizing carbon electrodes or TCO‐free designs typically use abundant materials which thereby reduces manufacture costs significantly [25]. While high‐throughput R2R printing is essential for enabling large‐volume production, system‐level analyses further showed that module manufacturing cost represents only a minor fraction of total installed system cost, with balance‐of‐system components dominating [77]. Lightweight and flexible perovskite modules can therefore access a wider commercial window by reducing installation costs, enabling competitiveness at lower efficiencies and shorter lifetimes than required for rigid modules. These studies indicate that the primary economic value of R2R perovskite technology lies in enabling simplified, low‐cost device architectures and lightweight form factors, rather than in web speed alone.

Beyond the cost and materials supply chain, solvent selection remains a key challenge in scaling R2R perovskite manufacturing due to its implications for safety, environmental impact, and process compatibility. Currently, most R2R demonstrations use N,N‐dimethylformamide (DMF) as the primary solvent for perovskite precursor solutions as demonstrated in Figure 6b. DMF is favoured for its strong solubility of lead halide salts and its ability to form uniform films at high coating speeds. However, it is also a reprotoxic solvent that has regulatory restrictions, with its toxicity raising safety concerns in large‐scale production environments. For instance, EU regulations now prohibit open use of DMF and impose strict controls on NMP [78], while US OSHA/NIOSH and state laws cap solvent exposures (DMF 10 ppm TWA, NMP 1 ppm TWA in CA) [79]. Robust engineering controls (closed‐loop recovery, ATEX gear) and solvent‐management systems are likely to be key enablers when manufacturing using high‐toxicity solvents. In contrast, alternatives like ethanol, isopropanol, ethyl acetate, anisole, GBL, and water‐based inks offer lower toxicity [80]. However, switching solvents often impacts film quality and drying kinetics. Ultimately, a combined strategy of regulatory compliance, safer solvent selection, and engineering controls is needed for sustainable roll‐to‐roll PSC manufacturing at scale [33]. Indeed, Wagner et al. estimate that producing 1 TWp of perovskite tandem modules would require the use of over 1 million metric tons of solvents annually if not recycled, with DMF and DMSO among the largest contributors. Solvent recovery and recycling systems, particularly in high‐volume production, are therefore essential. Achievable solvent recovery rates above 70% could significantly reduce net solvent consumption and associated emissions. Alternatively, less hazardous solvent systems such as alcohol‐based or green solvent formulations are currently being explored but have yet to demonstrate comparable performance in R2R processes [80]. Thus, while current R2R processes rely heavily on DMF‐based inks, transitioning to scalable, low‐toxicity solvent systems and implementing solvent recycling will be critical for sustainable high‐throughput manufacturing. Table 5 summarizes the challanges and corresponding mitigation strategies in R2R perovskite solar cell fabrication.

**TABLE 5:** Summary of Challenges and mitigation strategies in fabrication of R2R Perovskite Solar Cells.

Challenge	Main Issues	Mitigation Strategies / Approaches	Refs.
Controlled Film Formation	Rapid crystallisation on a moving substrate Non‐uniform grain formation	Gas quenching/vacuum quenching	[47, 55, 66]
		Solvent Engineering (volatile solvents)	[72]
		Additives (PEO and Cs salts) to tune crystallisation and surface tension	[30, 21]
		Hot casting/ substrate temperature control	[44]
Ambient processing	Moisture and oxygen sensitivity Degradation during coating outside inert environments	Humidity‐tolerant additives and two‐step deposition	[30, 57]
		Process optimisation via high‐throughput parameter mapping	[26]
Electrode deposition and module layout	High cost and vacuum processing of Au/Ag electrodes Complex patterning for series connection	Carbon‐based printable electrodes	[26, 27]
		ITO‐free and back‐contact device architectures	[25]
		Integration of inline laser or masked patterning for P1–P3 scribing	[26]
Scalability and cost	Material and solvent supply risks; expensive components (Au, ITO) Environmental and safety issues with toxic solvents	Substitution with earth‐abundant materials (Carbon, Cu, Al, AZO)	[26, 27, 76]
		Solvent recovery and recycling	[75]
		Low‐toxicity or green solvent systems	[80]
		Cost reduction via high‐throughput continuous R2R lines	[26]

## 5 Conclusions and Outlook

R2R fabrication is rapidly transforming perovskite solar cells from a lab curiosity into a scalable photovoltaic technology. Indeed, slot‐die coating provides a path to manufacture PSCs in a continuous, cost‐effective manner; features that are essential for low cost ($/W) mass production. Over the past few years, significant progress has been made in addressing the challenges of R2R PSC fabrication. The efficiencies of roll‐processed cells have increased from around 5% to 17% by using improved ink formulations and printing methods. Strategies such as printed carbon electrodes and optimized layer stacks have allowed fully printed devices to be realized. The stability of R2R printed PSCs is also improving through the use of more robust materials and encapsulation techniques, with promising news reported from industry. Various cost analysis studies indicate that printed PSC technology can be economically competitive, especially if expensive materials (such as gold electrodes or indium‐based TCOs) are replaced with low‐cost printed alternatives.

Challenges remain on the road to commercialization, notably ensuring multi‐decade durability and scaling production to millions of square meters. However, solutions are being actively being developed. Recent demonstrations of fully R2R‐fabricated perovskite modules under ambient conditions represent a landmark achievement, showing that high‐efficiency large‐area devices can be made using low‐cost printing methods.

In closing, the progress reviewed here underscores that roll‐to‐roll fabrication is not only feasible for perovskite solar cells but is maturing rapidly. In the coming years, R2R perovskite solar cells are likely to evolve into a mainstream photovoltaic technology, complementing conventional solar panels with lightweight, flexible, and low‐cost solar films.

## Funding

Engineering and Physical Sciences Research Council (EPSRC) grant EP/V027131/1 (High‐efficiency flexible and scalable halide‐perovskite solar modules).

## Conflicts of Interest

D.G.L. is co‐director of the company Ossila Ltd, which retails materials used in perovskite and photovoltaic research. F.J., N.S.H., J.A., and D.S. are research scientists at Power Roll Ltd, a company developing perovskite photovoltaic technology. Power Roll Ltd had no role in the preparation, analysis, or writing of this manuscript.

## References

[1] A. Kojima , K. Teshima , Y. Shirai , and T. Miyasaka , “Organometal Halide Perovskites as Visible‐Light Sensitizers for Photovoltaic Cells,” Journal of the American Chemical Society 131 (2009): 6050–6051. 19366264 10.1021/ja809598r

[2] National Renewable Energy Laboratory (NREL), Best Research‐Cell Efficiency Chart , National Laboratory of the Rockies, (2026), https://www.nlr.gov/pv/cell‐efficiency.

[3] M. A. Green , E. D. Dunlop , M. Yoshita , et al., “Solar Cell Efficiency Tables (Version 67),” Progress in Photovoltaics: Research and Applications 34 (2026): 482–496.

[4] N.‐G. Park , “Perovskite Solar Cells: An Emerging Photovoltaic Technology,” Materials Today 18 (2015): 65–72.

[5] G. W. P. Adhyaksa , L. W. Veldhuizen , Y. Kuang , S. Brittman , R. E. I. Schropp , and E. C. Garnett , “Carrier Diffusion Lengths in Hybrid Perovskites: Processing, Composition, Aging, and Surface Passivation Effects,” Chemistry of Materials 28 (2016): 5259–5263.

[6] G. Liu , M. Ghasemi , Q. Wei , B. Jia , Y. Yang , and X. Wen , “Dynamic Defect Tolerance in Metal Halide Perovskites: From Phenomena to Mechanism,” Advanced Energy Materials 15 (2025): 2405239.

[7] K. X. Steirer , P. Schulz , G. Teeter , et al., “Defect Tolerance in Methylammonium Lead Triiodide Perovskite,” ACS Energy Letters 1 (2016): 360–366.

[8] C. Yang , W. Hu , J. Liu , et al., “Achievements, Challenges, and Future Prospects for Industrialization of Perovskite Solar Cells,” Light: Science & Applications 13 (2024): 227. 10.1038/s41377-024-01461-x PMC11372181 39227394

[9] Q. Jiang and K. Zhu , “Rapid Advances Enabling High‐Performance Inverted Perovskite Solar Cells,” Nature Reviews Materials 9 (2024): 399–419.

[10] P. Docampo and T. Bein , “A Long‐Term View on Perovskite Optoelectronics,” Accounts of Chemical Research 49 (2016): 339–346. 26807593 10.1021/acs.accounts.5b00465

[11] M. H. Miah , M. U. Khandaker , M. B. Rahman , M. Nur‐E‐Alam , and M. A. Islam , “Bandgap Tuning of Perovskite Solar Cells for Enhancing the Efficiency and Stability: Issues and Prospects,” RSC Advances 14 (2024): 15876–15906. 38756852 10.1039/d4ra01640h PMC11097048

[12] L. A. Castriotta , M. A. Uddin , H. Jiao , and J. Huang , “Transition of Perovskite Solar Technologies to Being Flexible,” Advanced Materials 37 (2025): 2408036. 10.1002/adma.202408036 39817849

[13] Y. Hu , T. Niu , Y. Liu , et al., “Flexible Perovskite Solar Cells With High Power‐Per‐Weight: Progress, Application, and Perspectives,” ACS Energy Letters 6 (2021): 2917–2943.

[14] F. Song , D. Zheng , and J. Feng , “Mechanical Durability and Flexibility in Perovskite Photovoltaics: Advancements and Applications,” Advanced Materials 36 (2024): 2312041. 10.1002/adma.202312041 38219020

[15] H. J. Lomeri , A. Patra , G. Polino , et al., “Integration of a Paper‐Based Supercapacitor and Flexible Perovskite Mini‐Module: Toward Self‐Powered Portable and Wearable Electronics,” Advanced Functional Materials 34 (2024): 2313267.

[16] S. Aftab , G. Koyyada , Z. Ali , et al., “Flexible Perovskite Solar Cells: A Revolutionary Approach for Wearable Electronics and Sensors,” Materials Today Energy 51 (2025): 101872.

[17] T. M. Koh , H. Wang , Y. F. Ng , A. Bruno , S. Mhaisalkar , and N. Mathews , “Halide Perovskite Solar Cells for Building Integrated Photovoltaics: Transforming Building Façades Into Power Generators,” Advanced Materials 34 (2022): 2104661. 10.1002/adma.202104661 34699646

[18] E. Parvazian and T. Watson , “The Roll‐To‐Roll Revolution to Tackle the Industrial Leap for Perovskite Solar Cells,” Nature Communications 15 (2024): 3983. 10.1038/s41467-024-48518-4 PMC11088710 38734720

[19] D. I. Kutsarov , E. Rezaee , J. Lambert , W. T. Stroud , A. Panagiotopoulos , and S. R. P. Silva , “Progress in Flexible Perovskite Solar Cells: Paving the Way for Scalable Manufacturing,” Advanced Materials Technologies 10 (2025): 2401834.

[20] N. L. Chang , A. W. Y. Ho‐Baillie , D. Vak , M. Gao , M. A. Green , and R. J. Egan , “Manufacturing Cost and Market Potential Analysis of Demonstrated Roll‐To‐Roll Perovskite Photovoltaic Cell Processes,” Solar Energy Materials and Solar Cells 174 (2018): 314–324.

[21] G. Y. Park , M.‐J. Kim , J. Y. Oh , et al., “High‐Throughput Roll‐to‐Roll Processed Large‐Area Perovskite Solar Cells Using Rapid Radiation Annealing Technique,” ACS Applied Materials & Interfaces 16 (2024): 27410–27418. 38738751 10.1021/acsami.4c04153

[22] P. Holzhey , M. Prettl , S. Collavini , N. L. Chang , and M. Saliba , “Toward Commercialization With Lightweight, Flexible Perovskite Solar Cells for Residential Photovoltaics,” Joule 7 (2023): 257–271.

[23] E. Leccisi and V. Fthenakis , “Life Cycle Energy Demand and Carbon Emissions of Scalable Single‐Junction and Tandem Perovskite PV,” Progress in Photovoltaics: Research and Applications 29 (2021): 1078–1092.

[24] F. Jafarzadeh , L. Dong , D. Jang , et al., “Roll‐to‐Roll Deposition of Wide‐Bandgap CsFAPbBr_3_ Perovskite Solar Cells in Ambient Air With Optimized Ink Formulation,” Solar RRL 8 (2024): 2400530.

[25] D. Blackburn , N. S. Hill , C. J. Wood , et al., “Back‐Contact Perovskite Solar Cell Modules Fabricated via Roll‐to‐Roll Slot‐Die Coating: Scale‐Up Toward Manufacturing,” ACS Applied Energy Materials 8 (2025): 2219–2228. 40018388 10.1021/acsaem.4c02734 PMC11863245

[26] H. C. Weerasinghe , N. Macadam , J.‐E. Kim , et al., “The First Demonstration of Entirely Roll‐To‐Roll Fabricated Perovskite Solar Cell Modules Under Ambient Room Conditions,” Nature Communications 15 (2024): 1656. 10.1038/s41467-024-46016-1 PMC10933357 38472219

[27] D. Beynon , E. Parvazian , K. Hooper , et al., “All‐Printed Roll‐to‐Roll Perovskite Photovoltaics Enabled by Solution‐Processed Carbon Electrode,” Advanced Materials 35 (2023): 2208561. 10.1002/adma.202208561 36791080

[28] L. J. Sutherland , D. Vak , M. Gao , et al., “Vacuum‐Free and Solvent‐Free Deposition of Electrodes for Roll‐to‐Roll Fabricated Perovskite Solar Cells,” Advanced Energy Materials 12 (2022): 2202142.

[29] D. Burkitt , R. Patidar , P. Greenwood , et al., “Roll‐To‐Roll Slot‐Die Coated P–I–N Perovskite Solar Cells Using Acetonitrile Based Single Step Perovskite Solvent System,” Sustainable Energy & Fuels 4 (2020): 3340–3351.

[30] J. Kim , S. Kim , C. Zuo , M. Gao , D. Vak , and D. Kim , “Humidity‐Tolerant Roll‐to‐Roll Fabrication of Perovskite Solar Cells via Polymer‐Additive‐Assisted Hot Slot Die Deposition,” Advanced Functional Materials 29 (2019): 1809194.

[31] C. Zuo , D. Vak , D. Angmo , L. Ding , and M. Gao , “One‐Step Roll‐To‐Roll Air Processed High Efficiency Perovskite Solar Cells,” Nano Energy 46 (2018): 185–192.

[32] Y.‐J. Heo , J.‐E. Kim , H. Weerasinghe , et al., “Printing‐Friendly Sequential Deposition via Intra‐Additive Approach for Roll‐To‐Roll Process of Perovskite Solar Cells,” Nano Energy 41 (2017): 443–451.

[33] Y. Y. Kim , T.‐Y. Yang , R. Suhonen , et al., “Roll‐To‐Roll Gravure‐Printed Flexible Perovskite Solar Cells Using Eco‐Friendly Antisolvent Bathing With Wide Processing Window,” Nature Communications 11 (2020): 5146. 10.1038/s41467-020-18940-5 PMC7555830 33051454

[34] B. Roth , R. R. Søndergaard , and F. C. Krebs , Handbook of Flexible Organic Electronics. (Elsevier, 2015): 171–197.

[35] X. Ding , J. Liu , and T. A. L. Harris , “A Review of the Operating Limits in Slot Die Coating Processes,” AIChE Journal 62 (2016): 2508–2524.

[36] R. Keshavarzi , F. Hajisharifi , Z. Saki , et al., “Organic and Perovskite Solar Cells Based on Scalable Slot‐Die Coating Technique: Progress and Challenges,” Nano Today 61 (2025): 102600.

[37] S. P. Sutera and R. Skalak , “The History of Poiseuille's Law,” Annual Review of Fluid Mechanics 25 (1993): 1–20.

[38] J. P. Heller , “An Unmixing Demonstration,” American Journal of Physics 28 (1960): 348–353.

[39] P. W.‐K. Fong and G. Li , “The Challenge of Ambient Air–Processed Organometallic Halide Perovskite: Technology Transfer From Spin Coating to Meniscus Blade Coating of Perovskite Thin Films,” Frontiers in Materials 8 (2021): 635224.

[40] J. Lee , D. Lee , D. Jeong , and N. Park , “Control of Crystal Growth Toward Scalable Fabrication of Perovskite Solar Cells,” Advanced Functional Materials 29 (2019): 1807047.

[41] C. Liu , Y.‐B. Cheng , and Z. Ge , “Understanding of Perovskite Crystal Growth and Film Formation in Scalable Deposition Processes,” Chemical Society Reviews 49 (2020): 1653–1687. 32134426 10.1039/c9cs00711c

[42] X. Dai , Y. Deng , C. H. Van Brackle , and J. Huang , “Meniscus Fabrication of Halide Perovskite Thin Films at High Throughput for Large Area and Low‐Cost Solar Panels,” International Journal of Extreme Manufacturing 1 (2019): 022004.

[43] S. Siegrist , P. Nandi , R. K. Kothandaraman , A. Abdessalem , A. N. Tiwari , and F. Fu , “Understanding Coating Thickness and Uniformity of Blade‐Coated SnO_2_ Electron Transport Layer for Scalable Perovskite Solar Cells,” Solar RRL 7 (2023): 2300273.

[44] H. Li , C. Zuo , D. Angmo , H. Weerasinghe , M. Gao , and J. Yang , “Fully Roll‐to‐Roll Processed Efficient Perovskite Solar Cells via Precise Control on the Morphology of PbI_2_:CsI Layer,” Nano‐Micro Letters 14 (2022): 79. 35333995 10.1007/s40820-022-00815-7 PMC8956777

[45] M. Fievez , P. J. Singh Rana , T. M. Koh , et al., “Slot‐Die Coated Methylammonium‐Free Perovskite Solar Cells With 18% Efficiency,” Solar Energy Materials and Solar Cells 230 (2021): 111189.

[46] J. Li , J. Dagar , O. Shargaieva , et al., “Ink Design Enabling Slot‐Die Coated Perovskite Solar Cells With >22% Power Conversion Efficiency, Micro‐Modules, and 1 Year of Outdoor Performance Evaluation,” Advanced Energy Materials 13 (2023): 2203898.

[47] T. S. Le , D. Saranin , P. Gostishchev , et al., “All‐Slot‐Die‐Coated Inverted Perovskite Solar Cells in Ambient Conditions With Chlorine Additives,” Solar RRL 6 (2022): 2100807.

[48] B. Kang and F. Yan , “Emerging Strategies for the Large‐Scale Fabrication of Perovskite Solar Modules: From Design to Process,” Energy & Environmental Science 18 (2025): 3917–3954.

[49] S. Chen , X. Xiao , B. Chen , et al., “Crystallization in One‐Step Solution Deposition of Perovskite Films: Upward or Downward?,” Science Advances 2021, 7, abb2412. 10.1126/sciadv.abb2412 PMC10670903 33523938

[50] M. Abbas , L. Zeng , F. Guo , M. Rauf , X.‐C. Yuan , and B. Cai , “A Critical Review on Crystal Growth Techniques for Scalable Deposition of Photovoltaic Perovskite Thin Films,” Materials 13 (2020): 4851. 33138192 10.3390/ma13214851 PMC7663244

[51] Z. Yang , W. Zhang , S. Wu , et al., “Slot‐Die Coating Large‐Area Formamidinium‐Cesium Perovskite Film for Efficient and Stable Parallel Solar Module,” Science Advances 7 (2021): abg3749. 10.1126/sciadv.abg3749 PMC8087413 33931458

[52] M. Yang , Z. Li , M. O. Reese , et al., “Perovskite Ink With Wide Processing Window for Scalable High‐Efficiency Solar Cells,” Nature Energy 2 (2017): 17038.

[53] S. Chen , X. Dai , S. Xu , H. Jiao , L. Zhao , and J. Huang , “Stabilizing Perovskite‐Substrate Interfaces for High‐Performance Perovskite Modules,” Science 373 (2021): 902–907. 34413234 10.1126/science.abi6323

[54] S. Ternes , C. J. Brabec , L. A. Castriotta , et al., “Process Parameter Specification and Control in Solution Processing of Hybrid Perovskite Photovoltaics: From Domain‐Specific Jargon to Evidence‐Based, Unambiguous Description of Experimental Workflows,” Advanced Energy Materials 15 (2025): 03187.

[55] S. Ternes , F. Laufer , and U. W. Paetzold , “Modeling and Fundamental Dynamics of Vacuum, Gas, and Antisolvent Quenching for Scalable Perovskite Processes,” Advanced Science 11 (2024): 2308901. 38308172 10.1002/advs.202308901 PMC11005745

[56] M. Othman , F. Zheng , A. Seeber , et al., “Millimeter‐Sized Clusters of Triple Cation Perovskite Enables Highly Efficient and Reproducible Roll‐to‐Roll Fabricated Inverted Perovskite Solar Cells,” Advanced Functional Materials 32 (2022): 2110700.

[57] F. Jafarzadeh , L. Dong , D. Jang , et al., “Roll‐to‐Roll Deposition of Wide‐Bandgap CsFAPbBr_3_ Perovskite Solar Cells in Ambient Air With Optimized Ink Formulation,” Solar RRL 8 (2024): 2400530.

[58] Y. Xu , L. Zhu , J. Shi , et al., “The Effect of Humidity upon the Crystallization Process of Two‐Step Spin‐Coated Organic–Inorganic Perovskites,” Chemphyschem 17 (2016): 112–118. 26593743 10.1002/cphc.201500844

[59] G. A. Sepalage , H. Weerasinghe , N. Rai , et al., “Can Laminated Carbon Challenge Gold? Toward Universal, Scalable, and Low‐Cost Carbon Electrodes for Perovskite Solar Cells,” Advanced Materials Technologies 7 (2022): 2101148.

[60] B. Dou , J. B. Whitaker , K. Bruening , et al., “Roll‐to‐Roll Printing of Perovskite Solar Cells,” ACS Energy Letters 3 (2018): 2558–2565.

[61] Y. Galagan , F. Di Giacomo , H. Gorter , et al., “Roll‐to‐Roll Slot Die Coated Perovskite for Efficient Flexible Solar Cells,” Advanced Energy Materials 8 (2018): 1801935.

[62] Z. Gu , L. Zuo , T. T. Larsen‐Olsen , et al., “Interfacial Engineering of Self‐Assembled Monolayer Modified Semi‐Roll‐To‐Roll Planar Heterojunction Perovskite Solar Cells on Flexible Substrates,” Journal of Materials Chemistry A 3 (2015): 24254–24260.

[63] T. M. Schmidt , T. T. Larsen‐Olsen , J. E. Carlé , D. Angmo , and F. C. Krebs , “Upscaling of Perovskite Solar Cells: Fully Ambient Roll Processing of Flexible Perovskite Solar Cells With Printed Back Electrodes,” Advanced Energy Materials 5 (2015): 1500569.

[64] L. Fagiolari and F. Bella , “Carbon‐Based Materials for Stable, Cheaper and Large‐Scale Processable Perovskite Solar Cells,” Energy & Environmental Science 12 (2019): 3437–3472.

[65] S.‐H. Huang , K.‐Y. Tian , H.‐C. Huang , et al., “Controlling the Morphology and Interface of the Perovskite Layer for Scalable High‐Efficiency Solar Cells Fabricated Using Green Solvents and Blade Coating in an Ambient Environment,” ACS Applied Materials & Interfaces 12 (2020): 26041–26049. 32434322 10.1021/acsami.0c06211

[66] F. Jafarzadeh , L. A. Castriotta , F. De Rossi , et al., “All‐blade‐coated flexible perovskite solar cells & modules processed in air From a sustainable dimethyl sulfoxide (DMSO)‐based solvent system,” Sustainable Energy & Fuels 7 (2023): 2219–2228.

[67] J. Burschka , N. Pellet , S.‐J. Moon , et al., “Sequential Deposition as a Route to High‐Performance Perovskite‐Sensitized Solar Cells,” Nature 499 (2013): 316–319. 23842493 10.1038/nature12340

[68] F. Matteocci , L. Vesce , F. U. Kosasih , et al., “Fabrication and Morphological Characterization of High‐Efficiency Blade‐Coated Perovskite Solar Modules,” ACS Applied Materials & Interfaces 11 (2019): 25195–25204. 31268662 10.1021/acsami.9b05730

[69] C. Gong , S. Tong , K. Huang , et al., “Flexible Planar Heterojunction Perovskite Solar Cells Fabricated via Sequential Roll‐to‐Roll Microgravure Printing and Slot‐Die Coating Deposition,” Solar RRL 4 (2020): 1900204.

[70] L. A. Castriotta , M. Wang , X. Shi , B. D. Dou , L. T. Schelhas , and J. Huang , “Challenges, Technological Pathways and Trade‐Offs Of Perovskite Solar Modules for Long‐Term Operation,” Nat Energy (2026): 1–13.

[71] K. K. Shin Thant , C. Seriwattanachai , T. Jittham , N. Thamangraksat , P. Sakata , and P. Kanjanaboos , “Comprehensive Review on Slot‐Die‐Based Perovskite Photovoltaics: Mechanisms, Materials, Methods, and Marketability,” Advanced Energy Materials 15 (2025): 2403088.

[72] Y. Deng , C. H. Van Brackle , X. Dai , J. Zhao , B. Chen , and J. Huang , “Tailoring Solvent Coordination for High‐Speed, Room‐Temperature Blading of Perovskite Photovoltaic Films,” Science Advances 5 (2019): aax7537. 10.1126/sciadv.aax7537 PMC6897546 31840067

[73] J. Gong , S. B. Darling , and F. You , “Perovskite Photovoltaics: Life‐Cycle Assessment of Energy and Environmental Impacts,” Energy & Environmental Science 8 (2015): 1953–1968.

[74] T. Norgate and N. Haque , “Using Life Cycle Assessment to Evaluate Some Environmental Impacts of Gold Production,” Journal of Cleaner Production 29 (2012): 53–63.

[75] L. Wagner , J. Suo , B. Yang , et al., “The Resource Demands of Multi‐Terawatt‐Scale Perovskite Tandem Photovoltaics,” Joule 8 (2024): 1142–1160.

[76] J. W. C. Reinders , J. Bolding , C. Roldán‐Carmona , et al., “Room Temperature Pulsed Laser Deposition of Aluminum Zinc Oxide (AZO): Enabling Scalable Indium‐Free Transparent Conductive Oxides,” Advanced Functional Materials 35 (2025): 2418069.

[77] L. McGovern , E. C. Garnett , S. Veenstra , and B. Van Der Zwaan , “A Techno‐Economic Perspective on Rigid and Flexible Perovskite Solar Modules,” Sustainable Energy & Fuels 7 (2023): 5259–5270. 38013782 10.1039/d3se00828b PMC10596340

[78] Health and Safety Executive for Northern Ireland (HSENI), REACH—N,N‐Dimethylformamide Restriction from December 2023, https://www.hseni.gov.uk/articles/reach‐nn‐dimethylformamide‐restriction‐december‐2023, (accessed January 2026).

[79] Occupational Safety and Health Administration (OSHA), Dimethylformamide—Occupational Chemical Database, https://www.osha.gov/chemicaldata/481, (accessed January 2026).

[80] S. K. Podapangi , F. Jafarzadeh , S. Mattiello , et al., “Green Solvents, Materials, and Lead‐Free Semiconductors for Sustainable Fabrication of Perovskite Solar Cells,” RSC advances 13 (2023): 18165–18206. 37333793 10.1039/d3ra01692g PMC10269851

